# *LIN28B* affects gene expression at the hypothalamic-pituitary axis and serum testosterone levels

**DOI:** 10.1038/s41598-019-54475-6

**Published:** 2019-12-02

**Authors:** Jaakko T. Leinonen, Yu-Chia Chen, Jana Pennonen, Leevi Lehtonen, Nella Junna, Taru Tukiainen, Pertti Panula, Elisabeth Widén

**Affiliations:** 1 The Institute for Molecular Medicine Finland (FIMM), University of Helsinki, P.O. Box 20, Tukholmankatu 8, Helsinki, 00014 Finland; 2 Department of Anatomy and Neuroscience Center, University of Helsinki, P.O. Box 5 63, Haartmaninkatu 8, Helsinki, 00014 Finland

**Keywords:** Genetics, Transgenic organisms, Computational biology and bioinformatics, Heritable quantitative trait

## Abstract

Genome-wide association studies (GWAS) have recurrently associated sequence variation nearby *LIN28B* with pubertal timing, growth and disease. However, the biology linking *LIN28B* with these traits is still poorly understood. With our study, we sought to elucidate the mechanisms behind the *LIN28B* associations, with a special focus on studying *LIN28B* function at the hypothalamic-pituitary (HP) axis that is ultimately responsible for pubertal onset. Using CRISPR-Cas9 technology, we first generated *lin28b* knockout (KO) zebrafish. Compared to controls, the *lin28b* KO fish showed both accelerated growth tempo, reduced adult size and increased expression of mitochondrial genes during larval development. Importantly, data from the knockout zebrafish models and adult humans imply that *LIN28B* expression has potential to affect gene expression in the HP axis. Specifically, our results suggest that *LIN28B* expression correlates positively with the expression of *ESR1* in the hypothalamus and *POMC* in the pituitary. Moreover, we show how the pubertal timing advancing allele (T) for rs7759938 at the *LIN28B* locus associates with higher testosterone levels in the UK Biobank data. Overall, we provide novel evidence that *LIN28B* contributes to the regulation of sex hormone pathways, which might help explain why the gene associates with several distinct traits.

## Introduction

Pubertal timing is a complex trait that varies extensively in the general population. Individuals at the extreme tails of the distribution show an increased risk for many disease and socially adverse consequences including type 2 diabetes and depression^1^. Although several monogenic syndromes that affect pubertal timing have been identified, we still understand relatively little about the factors that control the timing of pubertal onset when development proceeds normally^2–5^. To shed more light on these mechanisms, our project focused on understanding the function of a pubertal timing-associated gene *LIN28B*, which has been robustly shown to affect developmental timing and growth in several species^6–8^.

While high-impact mutations causing rare monogenic forms of disturbed pubertal timing have been identified already a few decades ago, *LIN28B* presents one of the first genetic loci associated with puberty in genome-wide association studies (GWAS), affecting pubertal timing in the general population^9,10^. Recently, we showed that the same genetic variants nearby *LIN28B *that delay pubertal onset, also associate with increased *LIN28B* expression in the hypothalamus and the pituitary, the key structures regulating sexual maturation^11^. Since the original GWAS-studies linking *LIN28B* to puberty, the pleiotropic potential of the gene has become evident: currently, in humans, variants in the *LIN28B* region have been associated with several hormonally regulated traits ranging from depression and bone mineral density (BMD) to finger length ratios and height^12–15^.

Potentially, the pleiotropic effects of *LIN28B* might be a consequence of the gene’s local actions in different tissues during development, might occur due to systemic effects induced by the gene’s expression in the hypothalamus and the pituitary, or be a combination of both. At the molecular level, the *LIN28*-genes are known to control cell division, growth and differentiation - mainly through the well-established interaction with let7 microRNAs^16,17^. Many models suggest *LIN28B* and a homologous gene *LIN28A* contribute to cellular metabolism and proliferation: for example, both genes have been robustly implicated in several cancers and shown to affect metabolism in cells and mice^18,19^. Since *LIN28B* expression promotes cell proliferation, it is not surprising that the gene is both ubiquitously and prominently expressed during embryogenesis^20,21^. However, data from adult humans indicate that the genetic variants linked to pubertal timing affect *LIN28B* expression mainly in the hypothalamus and the pituitary, thus indicating that *LIN28B* primarily may impact on pubertal timing and related phenotypes through these tissues^11^.

The development of the hypothalamic-pituitary (HP) axis is well-conserved in vertebrates^22^. In this study, we have combined zebrafish knockout models with human data to address the genetic mechanisms whereby *LIN28B* might affect growth and development, with a special focus on *LIN28B* function at the HP axis. We demonstrate that in zebrafish CRISPR-Cas9 knockdown of *lin28b* accelerates the tempo of fish growth, simultaneously reducing the size of adult fish. Moreover, the *lin28b* KO zebrafish show evidence of altered expression of mitochondrial genes during larval development, as well as changes in estrogen signalling. We also show that increased *LIN28B* expression correlates with increased expression of several genes in the hypothalamus and the pituitary in humans, including *ESR1* and *POMC*. Finally, we report how the pubertal timing advancing alleles in the *LIN28B* locus associate with higher serum testosterone levels in the UK Biobank data, highlighting a general mechanism whereby *LIN28B* could affect several of the phenotypes associated with the gene.

## Results

### Generation of the *lin28b* KO zebrafish

**Figure 1:**
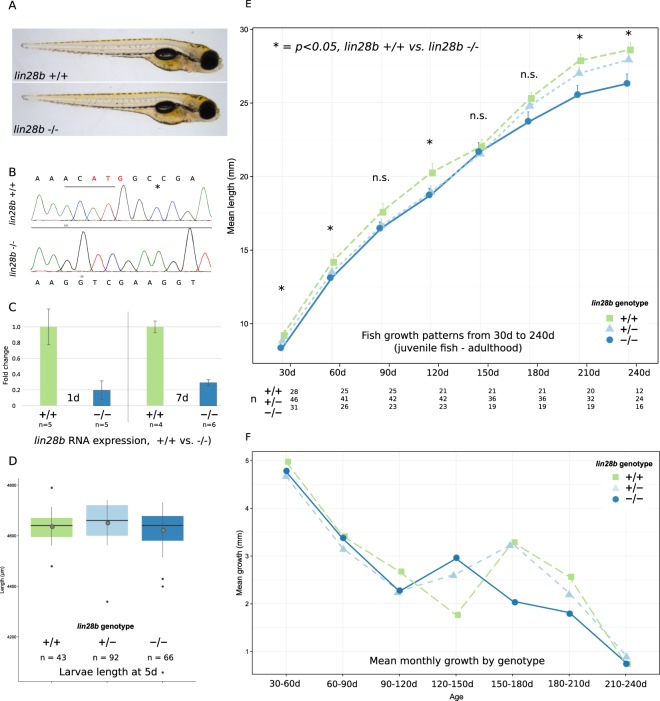
Characteristics and growth patterns of the *lin28b* KO fish. (**A**) Images of 5d old *lin28b* control (+/+) and KO (−/−) larvae (**B**) Sequencing traces showing the four deleted bases (underlined) + one mismatch (asterisk) in *exon1* mutant fish. The bases deleted in the mutants overlap the translation initiation codon for *lin28b* (in red). (**C**) Full length *lin28b* mRNA expression in control vs *lin28b* KO RNA-Seq samples at 1d and 7d. The expression of *lin28b* RNA appears downregulated in the KO fish, likely due to NMD related mechanisms. (**D**) Comparison of the *lin28b* KO and the control zebrafish size at 5d. No significant size differences were observed at this stage (One-Way-ANOVA with post-hoc Tukey HSD, Error bars = SEM). (**E**) Growth curves for the *lin28b* KO and the control fish. The growth of the KO fish was reduced, but the size difference temporarily vanishedaround the time of sexual maturation (One-Way-ANOVA with post-hoc Tukey HSD, Error bars = SEM, *P < 0.05). (**F**) Mean monthly growth by genotype. Around the period when the fish undergo sexual maturation the growth rate peaks transiently. The *lin28b* KO fish seem to undergo this transient peak in growth earlier than controls.

In order to assess the impacts of permanent *lin28b* dysregulation on zebrafish development, we generated *lin28b* mutant zebrafish strains with the help of CRISPR-Cas9 technology^23^. To create mutants by inducing double stranded breaks (DSB) into DNA, we designed guide RNA’s targeting exon1 and exon3 of zebrafish *lin28b* and co-injected these with Cas9 mRNA into embryos, resulting in mosaic fish (F0). These fish were subsequently outcrossed with the in-house strain, and the resulting offspring (F1) was screened for potential protein truncating mutations in *lin28b*. The generated protein truncating mutations that were chosen for this study either deleted the initiation codon (exon1) or introduced a premature stop codon to the canonical zebrafish *lin28b* transcript (exon3) (Supplementary data). Finally, two heterozygous fish were bred together to generate *lin28b* homozygous fish and their sibling controls (F2). The fish from these breedings generally showed the expected 1:2:1 genotypic ratio. However, the sex ratio of the fish appeared consistently distorted: despite the genotype of the F2 fish, the great majority of the fish (~85–90%) were male. As the sex determination in zebrafish appears complex, the result may reflect the fact that the fish which were used in this study were enriched for genetic factors promoting male development of that the growth conditions favoured the development of male fish. Hence, our results are largely based on data from male fish. Both exon1 and exon3 mutants were viable in the homozygous state, and showed no obvious phenotypes upon superficial visual inspection, despite a significant decrease in the amount of *lin28b* mRNA compared to controls (Fig. 1A–C). Since the different mutational strains were phenotypically indistinguishable, both exon1 and exon3 mutants (collectively referred to as the *lin28b* KO zebrafish) were used in parallel to produce data for the study. cDNA sequencing further confirmed *lin28b* RNA expressed in the mutants likely yields truncated protein that is prone to undergo non-sense mediated decay (NMD) (Supplementary data 1 and Supplementary Fig. 1).

### The *lin28b* KO fish show both advanced growth tempo and reduced growth

The *LIN28* gene family has been robustly associated with vertebrate growth, as the genes have been shown to contribute to the tempo and amplitude of growth in various species ranging from humans to nematodes. Previously, we analysed zebrafish models where *lin28b* was either transiently knocked down or overexpressed during the embryogenesis, showing how short-term, non-physiological *lin28b* overexpression can increase both larval and adulthood fish length^11^. In the current study, we wanted to test the consequences of permanent *lin28b* KO on fish growth and assessed these effects over the fish life-course from larval stages to adulthood.

We began by assessing whether the *lin28b* KO affected larval fish size, genotyping and measuring 5-day old zebrafish from heterozygote crosses (N = 201). However, at this stage, the length of the KO zebrafish appeared not significantly reduced (−/−) compared to the heterozygotes (+/−) and the wild-type siblings (+/+) (Fig. 1D).

Secondly, targeting the growth between the juvenile period and adulthood, we wanted to assess whether the knockouts exhibited similar alterations in the tempo and amplitude of longitudinal growth as previously reported for humans and mice. We designed an experiment where we tracked the growth of F2 zebrafish from 30 days post fertilisation (dpf) (juvenile fish) into 240 dpf (adult fish) by 30d intervals (Fig. 1E). Already by 30 dpf, the *lin28b* KO fish appeared on average significantly smaller than their *lin28b *+/+ siblings (p < 0.05). The KO fish remained smaller also at the end of the experiment at 240 dpf, matching the results from humans and mice linking lower *LIN28B* expression with smaller adult size. Although on average the size of the heterozygote fish appeared to fall between the +/+ and −/− groups, suggesting additive genetic effects, this group did not show statistically significant differences in growth compared to the other genotypes at any time point.

Besides causing smaller juvenile and adult size (growth amplitude), tracking the growth between these two time points allowed us to observe how the growth progresses throughout this period (growth tempo). Data from humans indicates that the individuals carrying alleles associated with lower *LIN28B* expression are born slightly smaller and show reduced adult height. Nonetheless, since the timing of their pubertal growth spurt is advanced, they temporarily appear taller than their peers in their early teens^6^. In fish stocks, growth patterns between the individual fish are known to vary extensively, but similarly as in humans, before sexual maturation, many zebrafish seem to experience transient slowing of growth followed by a period of more rapid growth^24^. Remarkably, we observed a similar trend in the growth patterns of the *lin28b* KO fish as seen in the human data (Fig. 1F). Around the timing of sexual maturation, (120–150 dpf in our strain) the size difference between the *lin28b* KO and the control fish temporarily disappeared, only to reappear later. Thus, mirroring the growth patterns previously reported in humans, our data suggest that in addition to affecting zebrafish size, *lin28b* may regulate the temporal pattern of fish growth, with the *lin28b* KO advancing the growth velocity around the timing of sexual maturation.

### The *lin28b* KO fish show advanced HP axis development, links to estrogen signalling and upregulation of mitochondrial pathways

**Figure 2:**
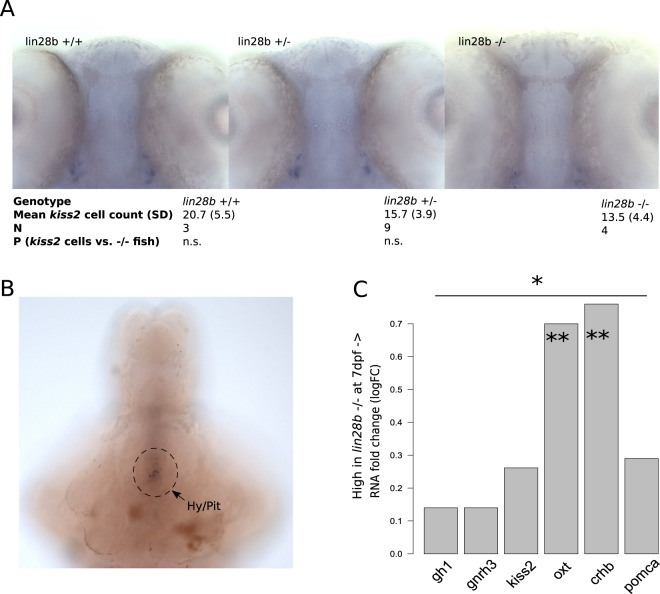
Evaluation of the *lin28b* KO effects on the HP axis in zebrafish larvae. (**A**) Representative images of *kiss2* expression at 3 dpf in the control (+/+), the heterozygous (+/−), and the *lin28b* KO (−/−) zebrafish. Though this varied between individual samples, the staining location and intensity was comparable between the groups. Despite a trend towards less *kiss2* expressing cells in the KO vs. control fish, we did not detect significant differences in the neuron numbers between the samples (Welch Two Sample t-test). The analysis was complicated by the fact that the +/+ group consists of WT fish from the exon3 crosses, whereas 3 out of 4 −/− fish were obtained from exon1 crosses. As the gene expression data from other experiments indicated that *kiss2* transcripts are not significantly up- or downregulated in the KO fish, we concluded that the *lin28b* KO does not prevent normal development of the *kiss2* neurons (e.g. **C**, Supplementary data 2, and Fig. 3). (**B**) Image of 7 dpf +/+ zebrafish brain showing weak staining by *lin28b* anti-sense mRNA in the hypothalamic-pituitary region. (**C**) HP axis RNA expression was slightly upregulated in the KO fish at 7 dpf. The analysis was restricted to hormonal genes that were expressed in high enough quantities to pass filtering in the RNA-seq experiment. Oxytocin (*oxt*) and corticotrophin-releasing-hormone beta (*chrb*) appeared upregulated in the KO samples (**p < 0.005, and overall the expression of the studied HP axis genes appeared upregulated in the *lin28b* KO zebrafish based on the RNAseq data from 7 dpf (*p < 0.05) (Welch two-sample t-test).

After discovering that our zebrafish model replicated some of the growth phenotypes previously associated with *LIN28B*, we turned our focus towards *lin28b* function at the HP axis. We first wanted to evaluate whether the *lin28b* KO fish showed changes in the structure and function of the HP axis already at the larval stages. We previously detected that knocking down *lin28b* temporarily in zebrafish embryos interfered with the development of the hypothalamic *kiss2* neurons, which are known to participate to gonadotropin release across species^11^. In the *lin28b* KO fish, the development of the *kiss2* neurons was however not dramatically affected at 3 dpf (Fig. 2A).

**Figure 3:**
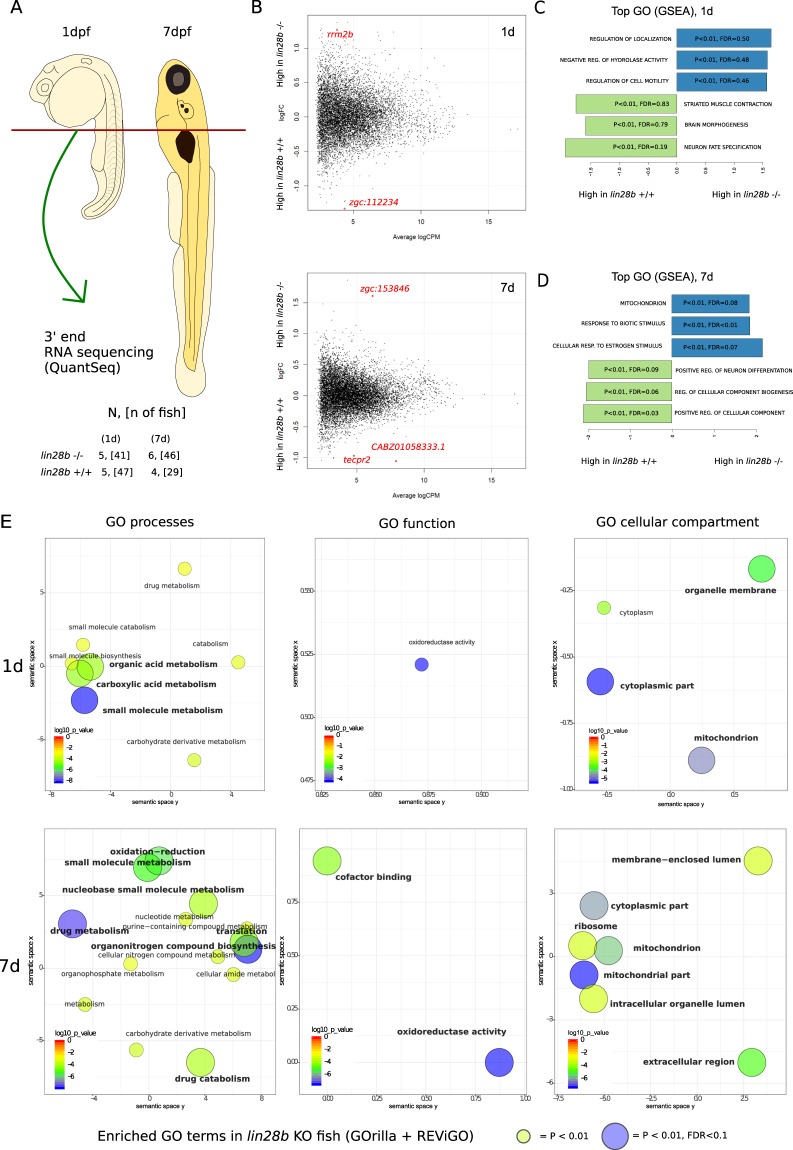
Summary of the RNA-Seq experiment examining the global RNA expression in the *lin28b* mutant fish at 1d and 7d. (**A**) Sampling and study design. After dissecting the anterior part from embryos/larvae, we pooled 6–10 samples together. The resulting 4–6 biological replicates per group were subjected to RNA sequencing. (**B**) Plots of mean logFC differences between the *lin28b* −/− and the +/+ fish at 1dpf (upper panel), and at 7dpf (lower panel). The genes showing significantly different expression in the global analysis (p < 0.00005, FDR < 0.1) are highlighted in red. (**C**,**D**) Results from the gene ontology analysis by GSEA, showing the GO categories differentiating between the *lin28b* −/− and the +/+ groups. E) Enriched GO terms in the *lin28b* −/− fish from the GOrilla analysis. Upper panels shows the results for the 1dpf fish and the lower panel for the 7dpf zebrafish.

To probe the HP axis development further, we performed RNA-seq analysis of embryonic and larval zebrafish. At 7 dpf, *lin28b* expression in the zebrafish brain seems already restricted to specific neuronal networks including the hypothalamus and pituitary (Fig. 2B)^11^. We first screened the RNA-seq data for expression of peptide hormones belonging to growth-hormone, gonadotropin, oxytocin or proopiomelanocortin pathways, and found out that six genes (*gh1*, *gnrh3*, *kiss2*, *oxt*, *crhb* and *pomca*) were expressed in high enough quantities to be subjected for detailed analysis. Although *kiss2* mRNA levels were not significantly affected, the *lin28b* KO larvae showed increased expression of *oxt* and *crhb* mRNAs *(P* < *0*.*005)* at 7 dpf. When analysing the expression of all the six HP axis genes together, we noticed that the expression of these genes was collectively upregulated in the KO zebrafish at 7 dpf (*P* < 0.05, Welch Two Sample t-test, Fig. 2C). Besides these links to the function of the HP axis, we however saw no evidence for example for steroidogenic enzymes being affected at this stage (Supplementary Table 1). We then turned in to assessing the global gene expression patterns between the *lin28b* KO and the control fish. In the global analysis of the RNA-seq data, only a couple of transcripts passed false discovery rate (FDR), potentially owing to a relatively small sample size (Fig. 3 and Supplementary Fig. 2). These included *rrm2b* and *zgc:153846* that were upregulated, and *zgc:112234*, *tecpr2* and *ENSDARG00000090575*, downregulated in the *lin28b* KO samples. The gene upregulated in 1-day-old knockouts, *rrm2b* is a part of the ribonucleotide reductase complex, regulating DNA repair and mtDNA synthesis in non-proliferating cells, whereas *zgc:153846*, upregulated in 7d KO larvae encodes for a lens protein, γ-Crystallin^25,26^. The genes downregulated in the knockouts include a gene encoding for a histone 2B *(zgc:112234*), another one involved in the regulation of autophagy (*tecpr2*) and finally a transcript that has been annotated as a *rnf213* orthologue (*ENSDARG00000090575)*^27^. However, the gene ontology (GO) analyses using GSEA (Gene Set Enrichment Analysis) and GOrilla (Gene Ontology enRIchment anaLysis and visualization) highlighted several interesting pathways that appeared to differ between the KOs and controls^28,29^. In the GSEA analysis the top GO terms enriched for the *lin28b* KOs at 7 dpf included “mitochondrion”, “response to biotic stimulus” and “cellular response to estrogen stimulus” (*P* < 0.01 and FDR < 0.1), and the GOrilla results also highlighted the mitochondrial pathways (Fig. 3).

**Figure 4:**
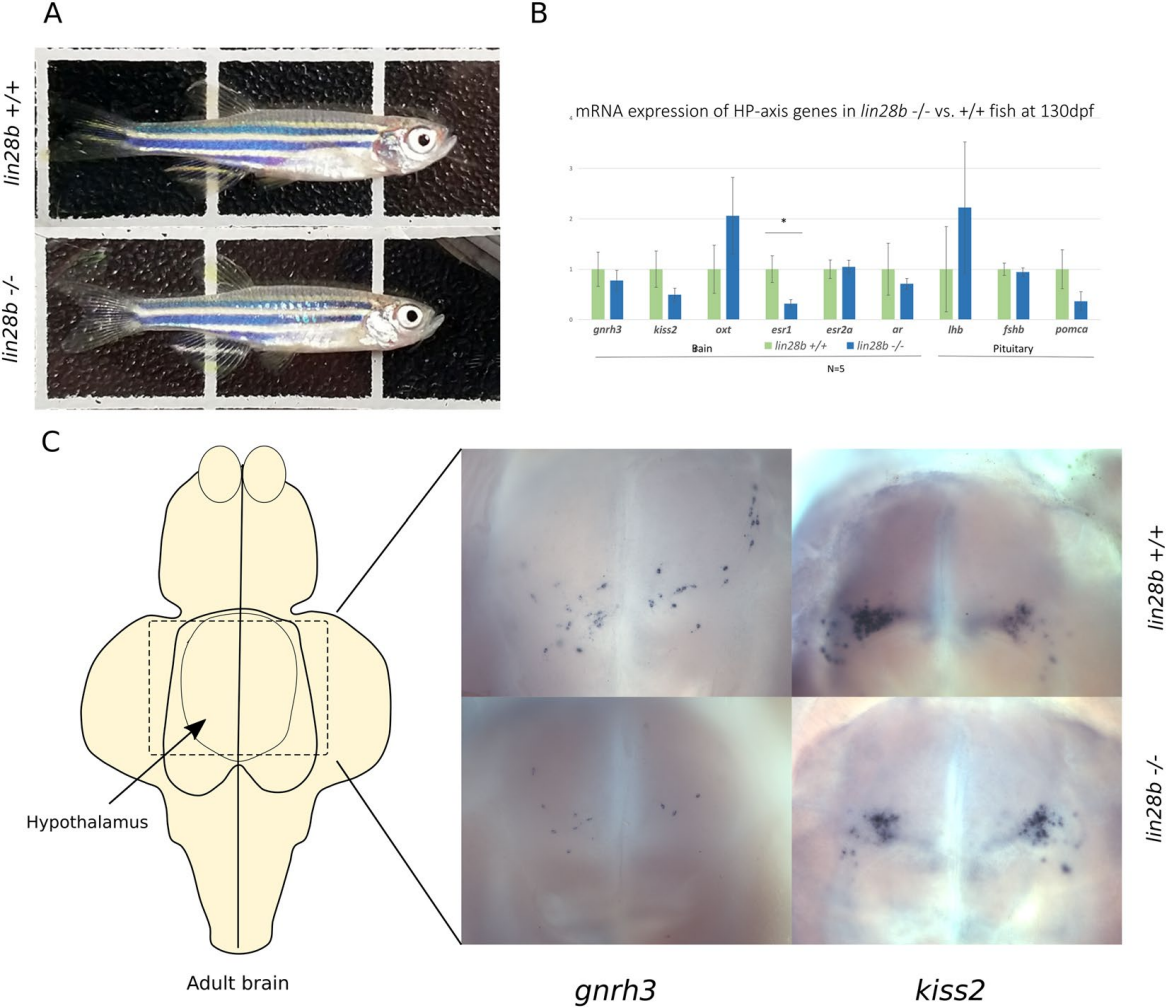
Inspection of the HP axis of the *lin28b* KO fish after sexual maturation. (**A**) Images of 130 day old fish (*lin28b* KO =−/−, lower panel). The KO fish behaved normally and showed no obvious phenotypic differences compared to controls at this stage. (**B**) qPCR results from 130d old fish evaluating gene expression at the HP axis. The results suggest that *esr1* expression is downregulated in the KO brain compared to controls, although for example *gnrh3* expression was not significantly different between the groups in the analysis. N = 5 per group, One-Way-ANOVA with post-hoc Tukey HSD, Error bars = SEM, *P < 0.05. Size of the fish used in the qPCR experiment is shown in the Supplementary Table 2. (**C**) Left: Schematic diagram of adult zebrafish brain (ventral view) with the hypothalamic region in the middle (arrow). Right: visualization of *gnrh3* and *kiss2* expression in the hypothalami of 210d old *lin28b* KO (−/−) and control (+/+) fish by *in situ* hybridization. The localization of *gnrh3/kiss2* expression appeared roughly similar, although the number of stained neurons varied between individual samples within each group (2–4 samples per group). The variation appeared potentially related to the size differences of the fish (for samples shown: *gnrh3*, +/+ size = 32 mm/300 mg; −/−, 27 mm/200 mg; *kiss2*+/+30 mm/230 mg, −/− 29 mm/190 mg).

After screening the RNA-seq data, to see whether the potential changes in the HP axis gene expression and estrogen signalling might last till adulthood, we then assessed the expression levels of estrogen signalling- and pubertal timing-related genes in the KO and control fish at 130 dpf by qPCR. We observed that *esr1* expression appeared downregulated in the KO-fish brain compared to the control fish (*P* = 0.04) (Fig. 4B). Although the expression of *pomca*, *gnrh3 and kiss2* also were on average lower in the *lin28b* KO fish than the controls, the differences were not statistically significant. Finally, we estimated whether the *lin28b* KO would impose changes in the organisation of hypothalamic Kiss2 or Gnrh3 neurons in adult fish. With *in situ* hybridisation we located respective RNA expression in adult *lin28b* KO and control zebrafish brains. We however could not detect any consistent differences in the location or staining intensity of *kiss2* and *gnrh3* expressing neurons between the KO and control fish (Fig. 4C).

### *LIN28B* expression correlates positively with the expression of several HP axis genes, including *ESR1* and *POMC* in adult humans

**Figure 5:**
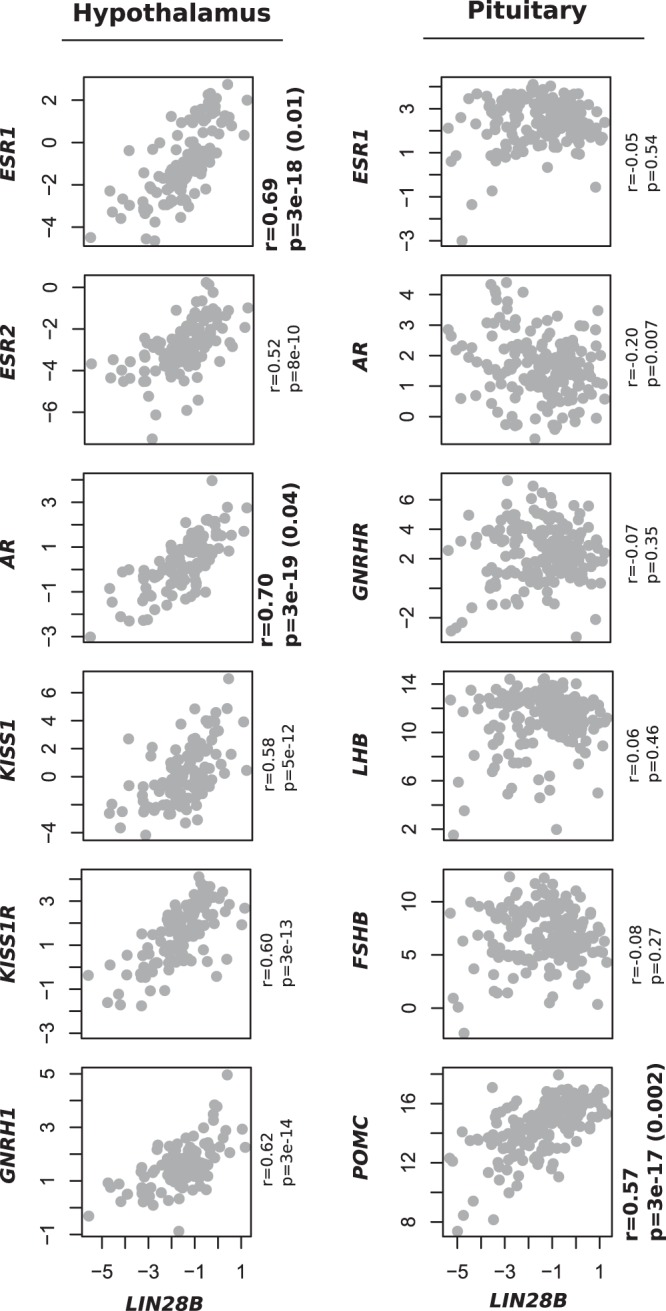
*LIN28B* correlations with hormonal genes in the hypothalamus and pituitary in GTEx. The figures show relative *LIN28B* expression on the X-axis compared to the expression of selected genes from the hypothalamus in the Y-axis (log2 TPM + 1). *LIN28B* expression correlated positively with all the tested genes in the hypothalamus (*R* = 0.58–0.70, *P* < 8e-10))). Particularly, the expression of *ESR1* and *AR* was upregulated: these genes showed statistically more robust correlations with *LIN28B* than the majority of the other ~50000 transcripts (p-value in parenthesis). Although *GNRHR*, *LHB* and *FSHB* showed no correlations with *LIN28B* levels in the pituitary, we observed a statistically significant positive correlation between *LIN28B* and *POMC* expression (*R* = 0.57, *P* = 3e-17). The correlation with *POMC* was among the most robust correlations for *LIN28B* in the pituitary.

The results from the *lin28b* KO fish prompted us to further assess the connection between *LIN28B*, estrogen signalling and HP axis function. In adult humans *LIN28B* expression is relatively strong in the hypothalamus and the pituitary compared to most tissues, and many of the phenotypes associated with *LIN28B* such as growth and puberty are regulated by the HP axis. Previous analyses by us and others further show that sequence variants in the *LIN28B* region affect the gene’s expression primarily in the hypothalamus and the pituitary, and a hypothesis has been proposed that decreasing *LIN28* expression in the hypothalamus might be permissive for pubertal onset^11,30^. To further explore *LIN28B* function at the HP axis, we leveraged the Genotype-Tissue Expression (GTEx) database, containing tissue specific gene expression data from hundreds of adult humans, looking for correlations between *LIN28B* and selected hormonal genes expressed in the HP axis (Fig. 5). The current release of GTEx (v7) consists of 11688 post mortem samples from mostly white adults, with median age over 50 yrs^31^. We included in this analysis pubertal timing genes belonging to kisspeptin-GNRH-gonadotropin pathway (*KISS*, *KISS1R*, *GNRH1*, *GNRHR*, *LH*, *FSHB*), and genes that were considered relevant based on the zebrafish data, including steroid hormone receptors that regulate the activity of pubertal timing pathway (*ESR1*, *ESR2*, *AR*) and proopiomelanocortin (*POMC*), the most abundant hormonal mRNA expressed in the pituitary. We observed that in the hypothalami of adult humans, higher *LIN28B* expression correlated with higher expression of *ESR1*, *ESR2*, *AR*, *KISS1*, *KISS1R* and *GNRH1* (unadjusted *P*-values ranging from 8e-10 to 3e-19). Compared to other genes expressed in the hypothalamus at a similar level, the correlation of *LIN28B* with *ESR1* and *AR* was among the top 5% of most significant results (permuted *P*-value < 0.05). Thus, contrasting our expectations, higher *LIN28B* expression in the hypothalamus, which previously has been linked with later pubertal onset, correlated with higher expression of genes that are known to promote sexual maturation.

The hypothalamic pathway including *KISS1* and *GNRH1* stimulates pituitary gonadotropin secretion through gonadotropin releasing hormone receptors (GNRHR). Therefore, we expected that higher *LIN28B *expression would correlate with higher mRNA levels of *GNRHR*, lutenizing hormone (*LH*) and follicle-stimulating hormone (*FSH*). Nonetheless, *GNRHR*, *LH* and *FSH* levels did not correlate with *LIN28B *expression in the pituitary (Fig. 5). Surprisingly, although *LIN28B* levels did not correlate with gonadotrophin mRNA expression, we noted that these correlated positively with the expression of *POMC*, coding a precursor for several peptides including b-endorphin, melanin, and adrenocorticotrophic hormone (ACTH) (unadjusted *P* = 3e-17, permuted *P* = 0.002, Fig. 5). While the current GTEx data include over 50,000 transcripts from the pituitary, we found that *POMC* was among the three most abundant transcripts in that tissue. Moreover, it was among the top 20 genes whose expression correlates with *LIN28B*^31^. The strong association with *POMC* further suggests that *LIN28B* may – either directly or indirectly – contribute to hormonal dynamics at the HP axis.

### Pubertal timing advancing alleles at the *LIN28B* locus associate with higher serum testosterone levels

Collectively, the results from our zebrafish models and the GTEx-database suggest that *LIN28B* may affect the expression of hormonal genes at the HP axis in an unexpected manner. Therefore, we finally wanted to explore the possibility that the gene might affect serum hormone levels in adults. Previously, we have shown that the puberty-delaying allele (C) at rs7759938 nearby *LIN28B* associates with higher *LIN28B* expression at the HP axis. For the current study, we performed an association analysis to detect whether the same variant would correlate with serum testosterone levels in the UK biobank database containing over 350,000 individuals with testosterone data available. Remarkably, we detected that the C-allele at rs7759938 was robustly associated with significantly lower serum testosterone levels (P = 9.2e-16, Table 1). The result was mostly driven by the signal in males (*P* = 2.5E-37) whereas it did not reach genome-wide significance (P < 5e-8) in females (*P* = 0.0067). The data, nonetheless, strongly link higher *LIN28B* expression with lower serum testosterone levels in adult humans.

**Table 1:** Association of rs7759938 with serum testosterone levels in the UK biobank data. A1 = effect allele, A2 = other allele, EAF = effect allele frequency.

	SNP	N	Chr	Pos	A1/A2	EAF	Beta	SE	P
Males	rs7759938	176212	6	105378954	C/T	0.32	−0.044	0.003	1.5E-37
Females	rs7759938	174850	6	105378954	C/T	0.32	−0.010	0.003	0.0067
M + F combined	rs7759938	351062	6	105378954	C/T	0.32	−0.020	0.002	9.2e-16

## Discussion

Over the past years, GWAS have linked sequence variants at the *LIN28B* locus with multiple traits. In the current study, we focused on exploring the mechanisms behind these associations. Our data show that *lin28b* affects growth and metabolism in zebrafish, and links the gene with estrogen signalling. Importantly, analyses of gene expression data from humans indicate that *LIN28B* may affect hormonal dynamics at the hypothalamus and pituitary, and based on data from ~350,000 individuals from the UK Biobank we report a novel association with *LIN28B* and serum testosterone levels. Altogether, these results provide important insights into the potential mechanisms whereby *LIN28B* may contribute to human phenotypes.

Despite the variable physical features of different vertebrates, many basic mechanisms regulating growth have been conserved during the course of evolution. For example, genetic variation in *VGLL3* has been shown to affect timing of sexual maturation in humans as well as in fish, and *LIN28* genes have been shown to contribute to growth and developmental timing in a wide range of metazoans ranging from *C*. *elegans* to humans^6,11,32–34^. In this study, we report that knocking out *lin28b* accelerates the growth tempo but leads to reduced adult size in zebrafish. The growth pattern of the *lin28b* KO zebrafish is strikingly similar to humans who carry SNPs that associate with lower *LIN28B* expression^6^. The result suggests that the *LIN28B*-related genetic mechanisms control ontogeny across different vertebrate species.

With our study, we specifically aimed to investigate whether *LIN28B* contributes to the development and function of the HP axis. Our results suggest that while the development of the HP axis might be slightly advanced by the *lin28b* KO, the gene is not essential for the formation of the HP axis in zebrafish. Since zebrafish have been shown to activate various compensatory mechanisms to ensure normal HP axis development, even when critical genes such as *kiss2* or *gnrh3* have been knocked out, the finding that the *lin28b* KO does not dramatically affect the development of the HP axis is not surprising^35,36^. In fact, gene loss by knockout may be generally quite well compensated in zebrafish, which potentially could explain the discrepancy between the current and our previous study where transient knockdown of *lin28b* with MOs significantly interfered with the development of the hypothalamic *kiss2* neurons^11,37,38^. For example, it remains to be studied to which extent the homologous gene *lin28a* might compensate for the loss of *lin28b*. However, in the current study we saw evidence that knocking out *lin28b* might induce subtle changes in hormonal signalling throughout development: genes which respond to estrogen signalling were upregulated in the KO larvae and *esr1a* appeared downregulated in the adult *lin28b* KO brains. In our study we did not assess whether these changes might be attributable for example to changes in the expression of the *let7* microRNAs, which are the known regulatory targets of the *LIN28*-genes.

Besides the links between *LIN28B* and estrogen signalling, our *lin28b* KO zebrafish showed an increased expression of mitochondrial genes during the first days of development. Loss of *LIN28* function has been shown to increase mitochondrial activity and prolong mitochondrial mRNA half-lives also in murine pluripotent stem cell models^39^. Since mitochondrial metabolism is the preferred means of energy production in differentiated cells, theoretically, this might be a reflection of the *lin28b* KO zebrafish having less dividing, and more differentiated cells than controls at this stage. As speculated in the case of *LIN28A* and mice, this could lead to a reduction in the total amount cells in these fish, providing a potential mechanistic explanation for the association between *LIN28B* and vertebrate body size^40^.

In addition to studying *lin28b* expression in zebrafish, we also leveraged the GTEx database and showed that *LIN28B* affects gene expression in the HP axis of adult humans. The finding that *LIN28B* correlates positively with genes such as *KISS1* and *GNRH1* was somewhat unexpected, since the activity of these genes is known to promote sexual maturation – a phenotype related to lower *LIN28B* expression. However, given that the *LIN28B* expression-increasing allele correlates negatively with serum testosterone levels, one may speculate that *LIN28B* indirectly, via reduced testosterone levels, might promote the expression of these steroid hormone receptors through allostatic regulation. Moreover, one may further speculate that sex hormones binding ER an AR, including testosterone, might be the potential mediators whereby *LIN28B* could affect many of the phenotypes associated with the gene, including for example pubertal timing, finger length ratios, and increased bone mineral density (BMD). Although our study does not directly address causation, considering the known functions of testosterone (promotion of pubertal development, contribution to finger length ratios, and increasing BMD), in theory we could assume that pubertal development might be triggered earlier, finger length ratios are likely masculinized and BMD increased for individuals carrying testosterone-increasing genetic variants in the *LIN28B* locus^14,41,42^. Nonetheless, the molecular mechanisms underlying the association between *LIN28B* and testosterone levels remain unknown and therefore warrant further studies.

Besides correlating with testosterone levels, we saw evidence that *LIN28B* may affect also estrogen signalling. While this might be directly related to changes in androgen levels, in theory estrogen might also have an independent effect on several *LIN28B* associated phenotypes. Studies in mice have shown that estrogen receptor alpha (ER*α*) signalling in *Kiss1* neurons is crucial for the regulation of pubertal onset: the ablation of *ERα* from these neurons dramatically advanced the pubertal development in mice, though stalling it later^43^. On the other hand, *ERα* knockout in medial basal hypothalamus leads to increased bone mineral density (BMD) in female mice via an effect on *Kiss1* neurons^44^. In principle, the positive correlation between *LIN28B* and *ESR1* expression in the hypothalamus thus might be directly relevant for example to *LIN28B* associations with puberty and BMD.

Further underscoring the widespread effects that *LIN28B* might have on hormonal genes expressed at the HP axis, we additionally observed a strong positive correlation between *LIN28B* and *POMC* expression in the pituitary. Notably, also increased *POMC*-activity has been associated with lower prenatal testosterone exposure^45^. *POMC* gives rise to several peptides that regulate human physiology, like β-endorphin, which might be relevant for example to *LIN28B* association with depression^46^. Overall, the results from our study suggest that *LIN28B* has widespread effects on gene expression at the HP axis, and *LIN28B*-induced changes in sex steroid signalling might contribute to many of the phenotypes associated with the gene in the human studies.

## Conclusion

Taken together, our results suggest that *LIN28B *genes regulate growth and development across vertebrates in an evolutionarily conserved manner. Besides showing how *lin28b* knockout affects zebrafish growth, our results indicate that *LIN28B* can contribute to gene expression dynamics at the HP axis, highlighting especially the *ESR1-* and *POMC-*related pathways. Importantly, the sequence variants linked to higher *LIN28B* expression also associate with lower serum testosterone levels in adult humans, which might mediate the gene’s association with various phenotypes.

## Materials and Methods

### Generation of the *lin28b* KO zebrafish

Zebrafish for the experiments were obtained from a breeding line that has been maintained in the Panula laboratory for more than 10 years (Kaslin and Panula, 2001; Kaslin *et al*., 2004). Permits for the experiments were attained from the Office of the Regional Government of Southern Finland, in agreement with the ethical guidelines of the European convention for the protection of vertebrate animals used for experimental and other scientific purposes.

We designed two CRISPR-Cas9 models targeting exon1 and exon3 of zebrafish *lin28b*. The target site for exon1 was 5′-GGAGGGTCCGCGGGGAAACA-3′ and for exon3 5′-GGCTTCAGGAGCCTGCGGGA-3′. To create mutants, 30 zebrafish embryos were injected with 600 pg Cas9 mRNA and 50 pg exon1 or 25 pg exon3 gRNA. (Supplementary Table 3). Mutations in *lin28b* were induced in approximately 80–90% of F0 fish. Fish from the two mutational strains, carrying mutations either in exon1 or exon3 of *lin28b* were mated with the in-house strain to obtain F1 zebrafish heterozygous for *lin28b* mutations. These two strains of F1 fish were subsequently crossed to obtain F2 control (+/+), heterozygous (+/−) and KO (−/−) zebrafish used in the analyses. Details of the induced mutations are shown in Supplementary data 1 and Supplementary Fig. 1.

### Fish growth analysis

We characterized the growth of the *lin28b* KO fish in two separate experiments. First, we targeted the larval fish size (at 5d) and in another experiment followed the growth of juvenile fish up to adulthood (from 30 dpf to 240 dpf). In the experiment including larval fish we measured altogether 201 5d old fish that were imaged, genotyped, and analyzed for their body length and head circumference. All the fish in this experiment were obtained from *lin28b* exon1 F1 crosses.

In the from-juvenile-to-adult experiment, fish length and weight were measured at 30d intervals (weight starting from 120dpf). Fish were genotyped from tail fin samples either by sequencing (exon1) or by High-Resolution-Melting curve analysis (HRM) (exon3) at 30d intervals. Sequencing and HRM primers and conditions are shown in Supplementary Table 3. *lin28b* +/+, +/− and −/− fish were grown together. Two of the tanks contained exon1 KOs and controls, (starting N = 32 and 34 fish per 5 liter tank), and one contained exon3 KOs and controls N = 38). The researcher measuring and sequencing the fish (JP) was always blinded for their genotype information.

### RNA isolation, RNAseq and qPCR

Total RNA was extracted using miRNeasy mini Kit (Qiagen Inc., Valencia, CA) according to the instructions of the manufacturer, For the RNA-seq experiment, we collected altogether 163 genotyped *lin28b* −/− and +/+ fish at 1dpf and 7 dpf. Pooled samples each containing RNA from 6–11 embryos were subsequently subjected to 3′ end RNA sequencing (Fig. 4A), producing an average read count of >10 million reads per sample. After quality control and filtering, the differential expression (DE) analyses included altogether 9304 and 11262 genes for 1dpf samples and 7 dpf samples, respectively (Supplementary data 1). P-value distribution of the data suggested that notable differences in the gene expression profiles between the *lin28b* KO and the control samples were likely to exist: however, only a couple of transcripts passed the false discovery rate (FDR) (Fig. 4B, Supplementary Fig. 3A and supplementary data 1). Out of five pooled 1d KO samples subjected to RNA-seq, two were based on exon1 mutant fish, and three were based on exon3 mutants. Out of six 7d KO samples, 3 were based on exon1 mutants and other 3 on exon3 mutants. The 1d and 7d samples subjected to RNA sequencing were DNAse treated, showing median RNA Integrity Number (RIN) > 9.5. Quality and quantity of the the extracted RNA samples were analyzed with 2100 Bioanalyzer RNA 6000 Nano Kit (Agilent, Santa Clara, CA, USA) and Qubit RNA BR kit (Thermo Fisher Scientific, Waltham, MA, USA). For genomic DNA contamination measurement, Qubit DNA BR kit (Thermo Fisher Scientific, Waltham, MA, USA) was used. mRNA libraries were prepared from 80 ng of extracted RNA with QuantSeq3′ mRNA-Seq Library Prep Kit (Lexogen GmbH, Vienna, Austria) according to user guide version 015UG009V0221. ERCC RNA spike-in mix (Life Technologies, Carlsbad, CA, USA) was added as control to each sample according to manufacturer’s instructions. Libraries were quantified and pooled for sequencing using 2100 Bioanalyzer DNA High Sensitivity Kit (Agilent, Santa Clara, CA, USA). Sequencing was performed with Illumina HiSeq2500 system in HiSeq high output mode using v4 chemistry (Illumina, San Diego, CA, USA). Read length for the paired-end run was 2 × 101 bp and target coverage 5 M reads for each library. Using Chipster analysis platform, sequencing reads were preprocessed with FastX and PRINSEQ, aligned to zebrafish genome build GRCz10 with TopHat2, and read count tables used in the analysis were produced by HTSeq. The final differential expression (DE) analysis between the KO and control samples was made in R version 3.4.3 using edgeR^47^. Gene ontology (GO) pathway analyses of the RNA-seq data were performed with Gene Set Enrichment Analysis (GSEA) and Gene Ontology enRIchment anaLysis and visuaLizAtion (GORILLA) tools in combination with REViGO^29,48^.

For qPCR experiments, RNA was extracted from a single brain/pituitary (130d). All samples in the 130 dpf experiment were obtained by breeding F1 *lin28b* exon1 mutants. 0.1–0.2 μg of RNA was reverse-transcribed using SuperScript^®^ VILO cDNA Synthesis Kit. The experiments were run with the Light Cycler^®^ 480 instrument using Roche Light Cycler 480 SYBR Green I master mix as instructed (F. Hoffmann-La Roche Ltd, Switzerland). 0.75 μl of 5 mM primers and 2 μl of 1:10 dilution of the cDNA was used per 15 μl reaction. Primer sequences and cycling conditions are shown in (Supplementary Table 3) Fluorescence changes were monitored after every cycle. Dissociation curve analysis was performed to ensure only a single amplicon was obtained. Reactions were run as duplicates and relative expression levels were calculated based on *b-actin,* using the 2-δδCT method.

### *In situ* hybridisation

WISH of *gnrh3* and *kiss2* probes was performed on 4% paraformaldehyde (PFA)-fixed brain from 240d old fish based on the protocol by Thisse & Thisse, as previously described^11^.

### GTEx

The Genotype-Tissue Expression (GTEx) project provides the scientific community with a resource of gene expression and genotype data from hundreds of individuals and thousands of samples from a diverse set of human tissues, with written informed consent for the use of samples obtained directly from the living participants, or their next-of-kin in case of deceased individuals^28^. We queried this database to examine whether *LIN28B* expression correlates with the expression of key genes involved in the regulation of pubertal timing in the hypothalamus and the pituitary.

For the current analysis release (GTEx Analysis V7 release, dbGaP Accession phs000424.v6.p1), the GTEx project has collected tissue samples from 714 postmortem donors (244 females, 470 males; age range 20–70), produced RNA sequencing data from 11688 tissue samples spanning 53 unique tissue types and generated genotype data for up to 635 donors. The data production and analysis procedures are available through the GTEx Portal (http://gtexportal.org) and described in detail in^28^.

### Genetic association analysis

For the current study, we utilised the UK Biobank database containing over 500,000 individuals, aged between 40 and 69 at the time of recruitment, described in detail by Bycroft *et al*.^49^. At recruitment, participants provided electronic signed consent. Ethics approval for the UK Biobank study was obtained from the North West Centre for Research Ethics Committee (11/NW/0382). All experiments were performed in accordance to relevant guidelines and regulations. Under UK Biobank application number 22627, we run an association analysis against serum testosterone for pubertal timing associated marker rs7759938, which was previously shown to correlate with *LIN28B* expression levels at the hypothalamus and the pituitary. Analysis was performed using linear mixed model association testing implemented in BOLT-LMM v2.3 software^50^. Sex-specific log-transformed testosterone levels were adjusted for age and BMI, inverse normalised, and the analyses were run using 10 PCs as covariates. Additional covariates included menopause for the analysis involving females and sex in the combined analysis. The analyses were restricted on individuals from white British ancestry, with testosterone levels between+/−5SD from the population mean for each sex.

### Statistical analyses

All analyses were performed with R version 3.4.3. Fish genotype ratios were assessed with Chi-square test. Growth of fish was compared with ANOVA using post-hoc Tukey HSD. Statistics for qPCR results from 130d fish were calculated using one-way ANOVA with post-hoc Tukey HSD.

## Supplementary information

Supplementary Dataset 1

Supplementary Dataset 2

## Data Availability

The gene expression datasets analysed during the current study are available from the GTEx portal (https://gtexportal.org/). The UK biobank data is available for researches upon request from the UK Biobank (https://www.ukbiobank.ac.uk/researchers/). The results from the RNA-seq dataset from 1d and 7d old zebrafish generated and analysed during the current study are included in the supplementary files.
