# Supplementary material for: LIN28B affects gene expression at the hypothalamic-pituitary axis and serum testosterone levels
Source: Sci Rep. 2019 Dec 2;9:18060. doi: 10.1038/s41598-019-54475-6 (PMC6889388; doi:10.1038/s41598-019-54475-6)
Supplement: Supplementary file 1 — Supplementary Dataset 1 [file 41598_2019_54475_MOESM1_ESM.pdf]

SUPPLEMENTARY FILE 1.

LIN28B affects gene expression at the hypothalamic-pituitary axis and serum testosterone levels

Jaakko T Leinonen, Yu-Chia Chen, Jana Pennonen, Leevi Lehtonen, Nella Junna, Taru Tukiainen, Pertti Panula and Elisabeth Widén

Supplementary data file 1.

A) 5’gatttcgctggaactttggaacggagggtccgcggggaaacATGGCCGAAG*GAGGGCCGCGCAGAGGTGGCGGGT
CGGACACCGCCAGGACTCCGCCGCAGAGTCTGTCTGGCTCGGGTTACTGCAAGTGGTTCAATGTCCGCATGGGGTTTGGA
TTTATATCGATGACCAGCAGCGAGGGGAAGCCAGTGGACCCTCCGCTAGACGTGTTCGTTCACCAAAGTAAGCTGGTGAT
GGAG*GGCTTCAGGAGCCTGCGGGAGGGCGAGCAGGTGGAGTTCACCTTTAAGAGGTCGAGTAAAGGTCTGGAGTCGCTC
CGGGTGACGGGGCCCGGGGGAGGCCCCTGCTCTGGCAGCGAGCGACGCCCCAAAGCAAAGGCCCCGCCCCTCAAACGCAA
ACCAAAGGGAGACCGGTGTATAACTGTGGAGGTCTGGACCACCACGCTAAAGAGTGTGGCCTTCCACCCCAGCCAAAGAA
GTGTCACTACTGTCAGAGTGTCACGCACATGGTGGCCCAGTGTCCCCACAAGGGGGCGCCGTCGCCCTCCGCGTCTCAGG
ACCCGCAACGCCCCTCCACCTCCGCTCAGTCCCCGGAAGAGGAAAGCCGCTCAGGCTCATCTTCATCCCCGGAGGAGGCT
TCTCAAAGAGGGAGTCGCTCCCAGCGCTGGAGAAAGAGCCGGGACTGAaacaaacacacctctcatccagaccaggcccg
gacacgaaatcatctacctcaaacag3’

B) 5’gatttcgctggaactttggaacggagggtccgcggggaa----GG**T**CGAAG*GAGGGCCGCGCAGAGGTGGCGGGT
CGGACACCGCCAGGACTCCGCCGCAGAGTCTGTCTGGCTCGGGTTACTGCAAGTGGTTCAATGTCCGCATGGGGTTTGGA
TTTATATCGATGACCAGCAGCGAGGGGAAGCCAGTGGACCCTCCGCTAGACGTGTTCGTTCACCAAAGTAAGCTGGTGAT
GGAG*GGCTTCAGGAGCCTGCGGGAGGGCGAGCAGGTGGAGTTCACCTTTAAGAGGTCGAGTAAAGGTCTGGAGTCGCTC
CGGGTGACGGGGCCCGGGGGAGGCCCCTGCTCTGGCAGCGAGCGACGCCCCAAAGCAAAGGCCCCGCCCCTCAAACGCAA
ACCAAAGGGAGACCGGTGTATAACTGTGGAGGTCTGGACCACCACGCTAAAGAGTGTGGCCTTCCACCCCAGCCAAAGAA
GTGTCACTACTGTCAGAGTGTCACGCAC**ATG**GTGGCCCAGTGTCCCCACAAGGGGGCGCCGTCGCCCTCCGCGTCTCAGG
ACCCGCAACGCCCCTCCACCTCCGCTCAGTCCCCGGAAGAGGAAAGCCGCTCAGGCTCATCTTCATCCCCGGAGGAGGCT
TCTCAAAGAGGGAGTCGCTCCCAGCGCTGGAGAAAGAGCCGGGACTGAaacaaacacacctctcatccagaccaggcccg
gacacgaaatcatctacctcaaacag3’

C) 5’gatttcgctggaactttggaacggagggtccgcggggaaacATGGCCGAAG*GAGGGCCGCGCAGAGGTGGCGGGT
CGGACACCGCCAGGACTCCGCCGCAGAGTCTGTCTGGCTCGGGTTACTGCAAGTGGTTCAATGTCCGCATGGGGTTTGGA
TTTATATCGATGACCAGCAGCGAGGGGAAGCCAGTGGACCCTCCGCTAGACGTGTTCGTTCACCAAAGTAAGCTGGTGAT
GGAG*GGCTTCAGGAG----------GGCGAGCAGGTGGAGTTCACCTTTAAGAGGTCGAGTAAAGGTCTGGAGTCGCTC
CGGG**TGA**CGGGGCCCGGGGGAGGCCCCTGCTCTGGCAGCGAGCGACGCCCCAAAGCAAAGGCCCCGCCCCTCAAACGCAA
ACCAAAGGGAGACCGGTGTATAACTGTGGAGGTCTGGACCACCACGCTAAAGAGTGTGGCCTTCCACCCCAGCCAAAGAA

GTGTCACTACTGTCAGAGTGTCACGCACATGGTGGCCCAGTGTCCCCACAAGGGGGCGCCGTCGCCCTCCGCGTCTCAGG
ACCCGCAACGCCCCTCCACCTCCGCTCAGTCCCCGGAAGAGGAAAGCCGCTCAGGCTCATCTTCATCCCCGGAGGAGGCT
TCTCAAAGAGGGAGTCGCTCCCAGCGCTGGAGAAAGAGCCGGGACTGAaacaaacacacctctcatccagaccaggcccg
gacacgaaatcatctacctcaaacag3’

D) 5’gatttcgctggaactttggaacggagggtccgcggggaaacATGGCCGAAG*GAGGGCCGCGCAGAGGTGGCGGGT
CGGACACCGCCAGGACTCCGCCGCAGAGTCTGTCTGGCTCGGGTTACTGCAAGTGGTTCAATGTCCGCATGGGGTTTGGA
TTTATATCGATGACCAGCAGCGAGGGGAAGCCAGTGGACCCTCCGCTAGACGTGTTCGTTCACCAAAGTAAGCTGGTGAT
GGAG*GGCTTCAGGAGCCT**TCAGGAGCCT***TCA*GGAGGGCGAGCAGGTGGAGTTCACCTT**TAA**GAGGTCGAGTAAAGGTCT
GGAGTCGCTCCGGGTGACGGGGCCCGGGGGAGGCCCCTGCTCTGGCAGCGAGCGACGCCCCAAAGCAAAGGCCCCGCCCC
TCAAACGCAAACCAAAGGGAGACCGGTGTATAACTGTGGAGGTCTGGACCACCACGCTAAAGAGTGTGGCCTTCCACCCC
AGCCAAAGAAGTGTCACTACTGTCAGAGTGTCACGCACATGGTGGCCCAGTGTCCCCACAAGGGGGCGCCGTCGCCCTCC
GCGTCTCAGGACCCGCAACGCCCCTCCACCTCCGCTCAGTCCCCGGAAGAGGAAAGCCGCTCAGGCTCATCTTCATCCCC
GGAGGAGGCTTCTCAAAGAGGGAGTCGCTCCCAGCGCTGGAGAAAGAGCCGGGACTGAaacaaacacacctctcatccag
accaggcccggacacgaaatcatctacctcaaacag3’

Supplementary data 1. Annotated zebrafish *lin28b* cDNA sequences showing the locations of CRISPR-Cas9 induced mutations.

A) Wild-type *lin28b* cDNA sequence. Primer locations shown in green, 5’ and 3’ UTR shown in lower case letters and exons in uppercase. * denotes exon boundary.

B) cDNA sequence for exon1 mutant fish, showing the deletion of four bases overlapping the translation intiation codon (ATG) and one mismatch (C>T). The only other potential intiation codon encoding for lin28b related transcript bolded and highlighted on red. The truncated protein potentially produced using this site would not contain the cold-shock and zinc-finger domains crucial for *lin28b* function.

C) cDNA sequence of exon3 mutant fish A, showing the deletion of 10 bases in *lin28b* exon 3. Premature stop codon bolded and highlighted in red.

D) cDNA sequence of exon3 mutant fish B, showing the insertion of 10 bases (red) + 3 mismatches (orange) compared to the wild-type sequence. Premature stop codon bolded and highlighted in red.

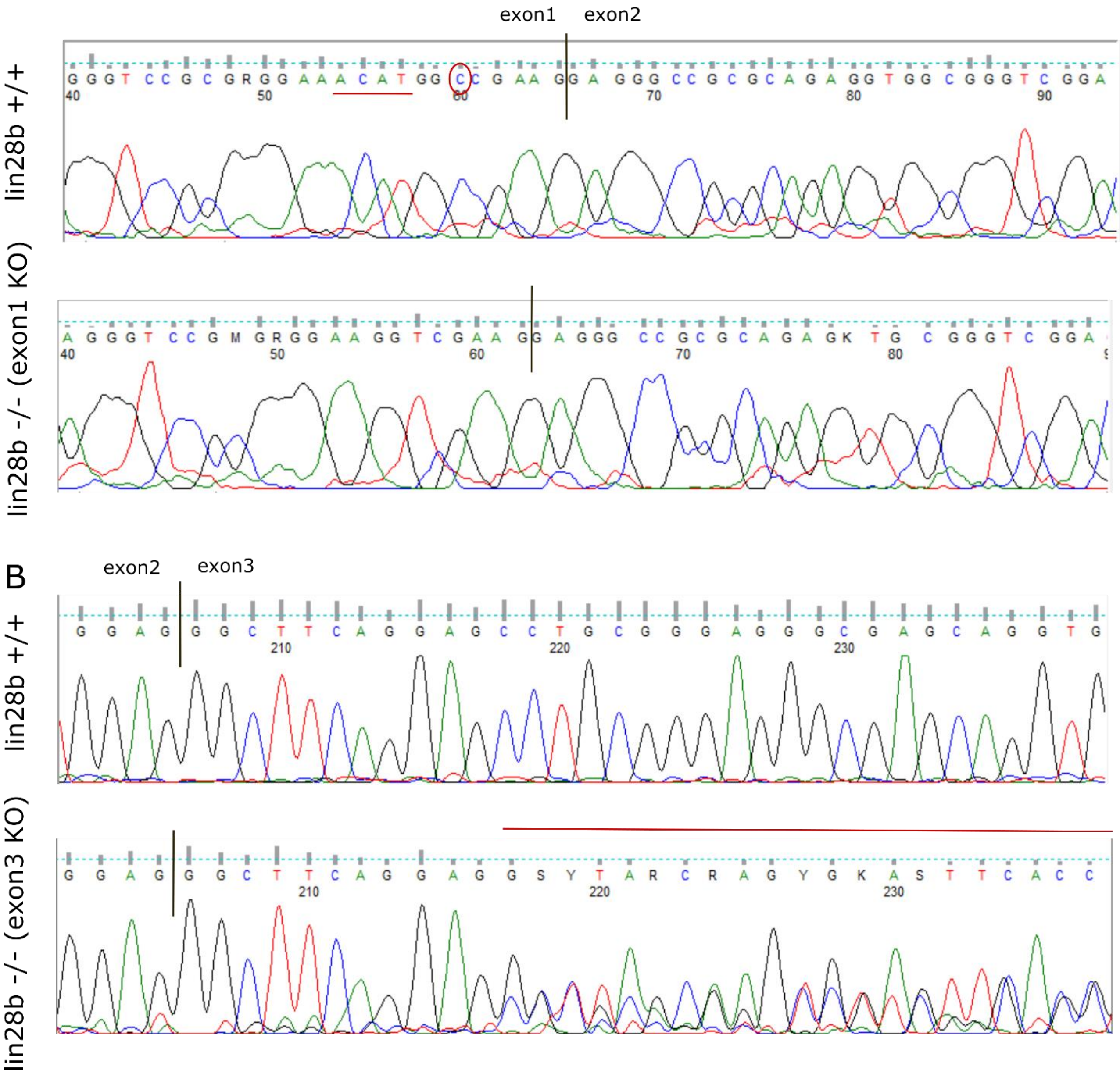

Supplementary figure 1. cDNA traces from *lin28b* control and KO fish.

A) Sequencing traces indicating the mutation location in exon1 mutant fish. Mutated based indicated with red lines and circle. Exon boundaries marked with black lines. The KO fish were homozygous for a exon1 mutation that deleted four base pairs and introduced one mismatch. B) Sequencing traces indicating the mutation location in exon3 mutant fish. Exon3 KO fish were compound heterozygous for exon3 mutations that either deleted 10 base pairs, or inserted 10 base pairs and 3 mismatches to the canonical *lin28b* cDNA sequence.

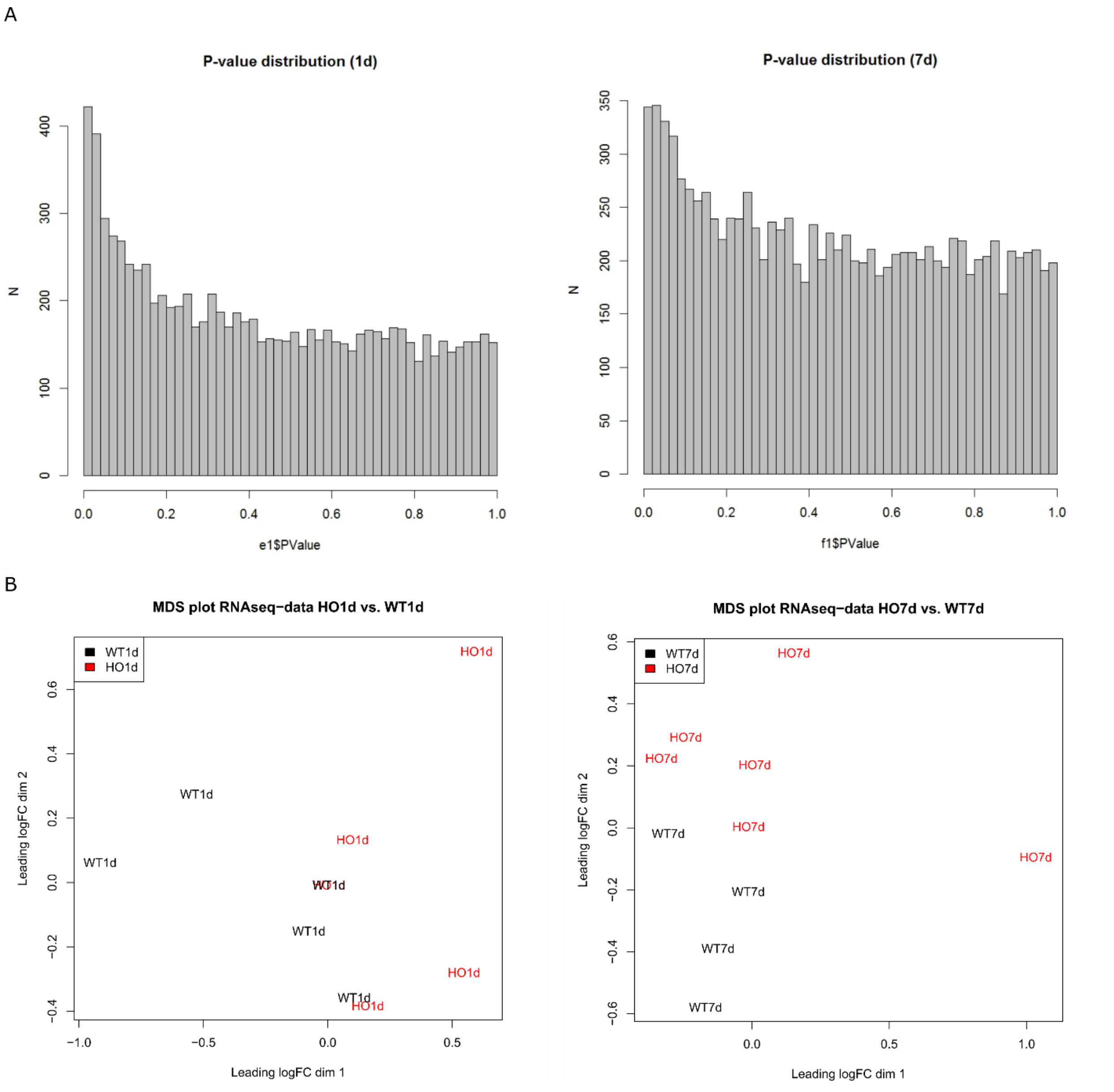

Supplementary figure 2. P-value distribution and MDS-plots from the RNA-seq experiment.

A) P-value distribution for 1d and 7d RNA-seq results, showing an excess of small p-values at both time points. The result suggest that more genes show differential expression (reflected by enrichment of small p-values) between the control and *lin28b* KO samples than expected by chance. X-axis shows the p-value divided into bins by 0.02 unit intervals, and Y-axis shows the number of genes in each bin. B) MDS-plots showing the clustering of the RNA-seq samples. At both time points (1d and 7d) the control (WT) and KO (HO) samples cluster separately, reflecting systematic differences in the gene expression between the groups.

Supplementary table 1. Analysis of differential expression of steroidogenic enzymes in 7dpf fish, based on RNA-seq data. None of the studied genes showed statistically significant differences between the +/+ and -/- groups. FC=Fold change, CPM=counts per million, FDR= False Discovery Rate.

Gene	logFC	logCPM	F	PValue	FDR
cyp11a1	2,15687	-1,0649	1,677746	0,195226	1
cyp11a2	0,014843	-0,86194	6,39E-05	0,993624	1
cyp11c1	-0,04601	0,666241	0,006203	0,937225	1
cyp17a1	0,14942	0,578306	0,067782	0,796632	0,954844
cyp17a2	1,657376	-1,25395	0,798593	0,371516	1
cyp19a1a	0,777384	-0,79322	0,36603	0,545177	1
cyp19a1b	0,569599	0,273617	0,696764	0,403875	1

Supplementary table 2. Characteristics of fish size included in the 130d qPCR experiment assessing HP axis gene expression in sexually mature zebrafish

Genotype	Length(mm)	Weight(mg)
KO	21,5	130
KO	21,5	140
KO	23	150
KO	24	170
KO	25	170
Control	22,5	140
Control	23	130
Control	23	170
Control	24	160
Control	25	180

Supplementary table 3. Primers and protocols used in the generation of lin28b KO zebrafish

	PCR primers for mutation sequencing	PCR cycling conditions for sequencing
exon1	F: 5'-TACAAACAACGTAAACAAAG-3'; R:5'-AAAGAGACATGACTAAATATC-3'	95°C 3min; 35x(95°C 30s, 55°C 30s, 72°C 1min); 72°C 10min
exon3	F: 5'-GACTCCAGACCTTTACTC-3'; R: 5'-TATACAAGAACGCCTGAT-3'	95°C 3min; 35x(95°C 30s, 55°C 30s, 72°C 1min); 72°C 10min
	HRM primers for mutation detection	HRM cycling conditions
exon1	NA	NA
exon3	F: 5'-CCTCTTAAAGGTGAACTCCA-3'; R:5'-GGGTGTAATAAATATACAAGAACG-3'	95°C 5min; 40x(95°C 30s, 55°C 30s, 72°C 30s); 72°C 10min
	Primers for *lin28b* amplification from cDNA	PCR cycling conditions for cDNA sequencing
	F:5'-GATTTCGCTGGAACTTTG-3'; R:5'CTGTTTGAGGTAGATGATTTC-3'	95°C 3min; 35x(95°C 20s, 55°C 20s, 72°C 30s); 72°C 10min
	guideRNA Target Sequence	
exon1	5’-GGAGGGTCCGCGGGGAAACA-3’	
exon3	5’-GGCTTCAGGAGCCTGCGGGA-3’	
	CRISPR-Cas9 Injection mixture *lin28b* exon1	CRISPR-Cas9 Injection mixture *lin28b* exon3
sgRNA exon1	0.5µl (195ng/µl)	0
sgRNA exon3	0	0.5µl (98ng/µl)
Cas9 mRNA	2µl (592ng/µl)	2µl (592ng/µl)
phenol red	0.7µl	0.7µl
H20	0.5µl	0.5µl
total	3.7µl	3.7µl
